# Probiotics in human gut microbiota can degrade host glycosaminoglycans

**DOI:** 10.1038/s41598-018-28886-w

**Published:** 2018-07-13

**Authors:** Keigo Kawai, Reiko Kamochi, Sayoko Oiki, Kousaku Murata, Wataru Hashimoto

**Affiliations:** 1 Laboratory of Basic and Applied Molecular Biotechnology, Division of Food Science and Biotechnology, Graduate School of Agriculture, Kyoto University, Uji, Kyoto, 611-0011 Japan; 2 Laboratory of Basic and Applied Molecular Biotechnology, Department of Food Science and Biotechnology, Faculty of Agriculture, Kyoto University, Uji, Kyoto, 611-0011 Japan; 3 Laboratory of Food Microbiology, Department of Life Science, Faculty of Science and Engineering, Setsunan University, Neyagawa, Osaka, 572-8508 Japan

## Abstract

Glycosaminoglycans (GAGs) (e.g. heparin, chondroitin sulfate, and hyaluronan) show various significant physiological functions as a major component of extracellular matrix in animals. Some bacteria target GAGs for adhesion and/or infection to host cells, although no probiotics have been known to degrade GAGs. Here, we show GAG degradation by probiotics from human gut microbiota and their adhesion to human intestinal cells through a GAG. GAG-degrading bacteria were isolated from human faeces and identified as *Enterococcus faecium*, and some typical probiotics such as *Lactobacillus casei*, *Lactobacillus rhamnosus* and *Enterococcus faecalis* were also found to degrade heparin. GAG-degrading lactobacilli and enterococci including the isolated *E. faecium* possessed a genetic cluster encoding GAG-degrading/metabolising enzymes in the bacterial genome. KduI and KduD enzymes encoded in the GAG cluster of *L*. *rhamnosus* functioned as 4-deoxy-l-*threo*-5-hexosulose-uronate ketol-isomerase and 2-keto-3-deoxy-d-gluconate dehydrogenase, respectively, both of which were crucial for GAG metabolism. GAG-degrading *L*. *rhamnosus* and *E*. *faecium* attached to human intestinal Caco-2 cells via heparin. Some species of *Bacteroides*, considered to be the next generation probiotics, degraded chondroitin sulfate C and hyaluronan, and genes coding for the *Bacteroides* GAG-degrading enzyme were frequently detected from human gut microbiota. This is the first report on GAG-degrading probiotics in human gut microbiota.

## Introduction

Animal cells are enveloped with an extracellular matrix constituted by a complex of supramolecules such as structural proteins, polysaccharides, and proteoglycans^1^. Glycosaminoglycans (GAGs), the major components of the extracellular matrix, are heteropolysaccharides constituted by the repetitive disaccharide units of uronic acid (or galactose) and amino sugar residues^2^. Based on their sugar composition, linkage mode of the sugar residues, and sulfation level, GAGs ubiquitously present in all mammalian tissues are classified mainly as hyaluronan, chondroitin sulfate, dermatan sulfate, heparan sulfate, heparin, or keratan sulfate (Fig. S1). Except for hyaluronan, these GAGs are covalently bound to core proteins, resulting in the formation of proteoglycans.

**Figure 1:**
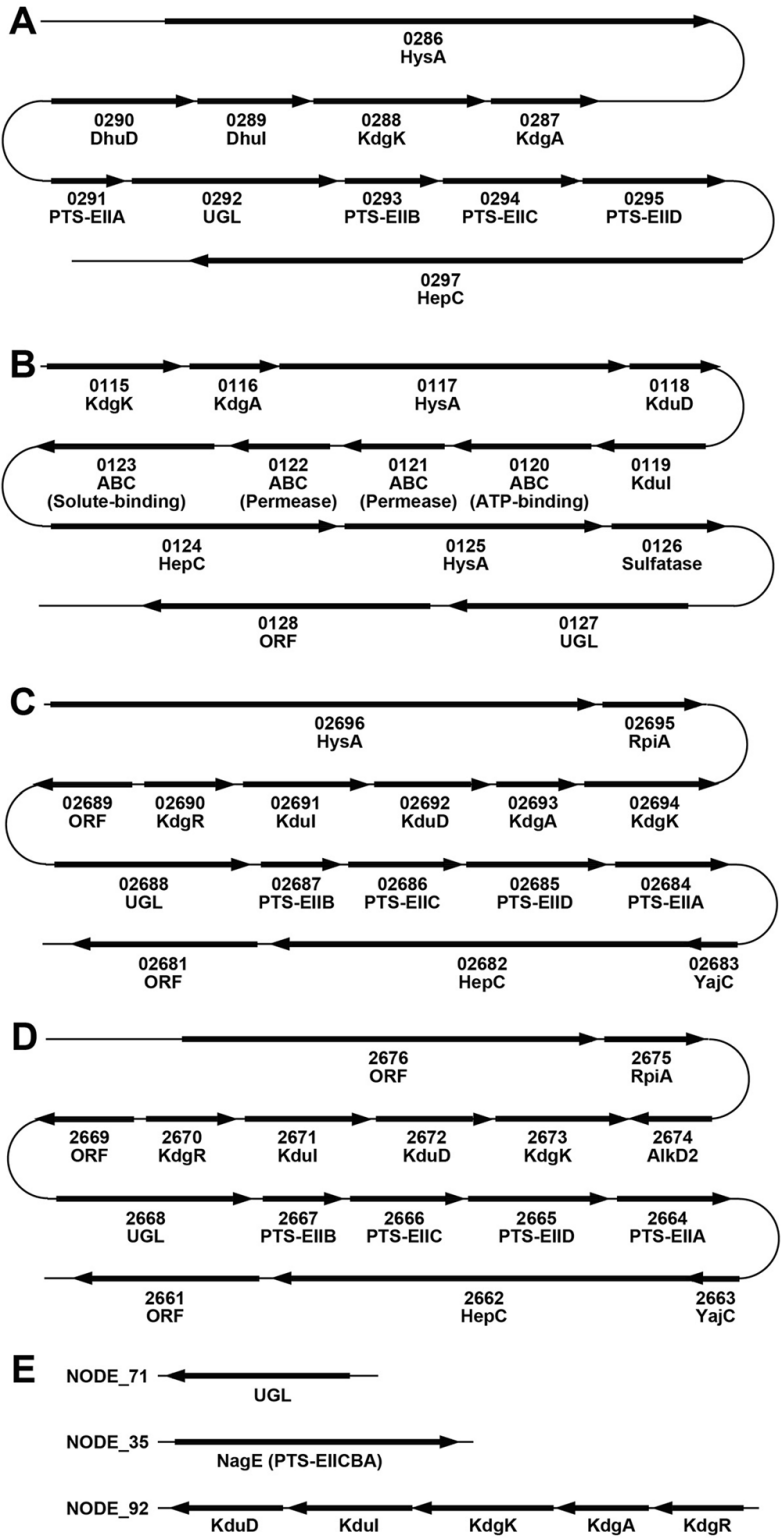
Varieties of bacterial GAG genetic clusters. (**A**) *Streptococcus* *pneumoniae* (strain R6). (**B**) *S*. *moniliformis*. (**C**) *L*. *rhamnosus* (strain Lc705). (**D**) *L. casei* subsp. *casei*. (**E**) *E. faecium* strain H57. Four or five digits indicate gene ID numbers of each bacterium (spr#### in *S*. *pneumoniae*, Smon_#### in *S*. *moniliformis*, Lc705_##### in *L*. *rhamnosus* and LSEI_#### in *L. casei* subsp. *casei*). Gene products are shown under gene ID. Isomerase and reductase similar to KduI and KduD for pectin metabolism, respectively, were encoded in the *Strepobacillus*, *Lactobacillus* and *Enterococcus* GAG clusters, while the *Streptococcus* cluster codes experimentally identified isomerase DhuI and reductase DhuD involved in GAG metabolism.

A large number of bacteria such as *Streptococcus*, *Listeria*, *Helicobacter*, *Enterococcus* and *Lactobacillus* target mammalian GAGs for colonisation and/or infection to host cells^3,4^. Among these, a few bacteria including *Streptococcus* are known to degrade GAGs^5,6^, although GAG binding by GAG-degrading bacteria such as *Bacteroides* and *Clostridium*^7^ remains to be clarified. In the case of *Streptococcus*, hyaluronan is depolymerised to an unsaturated disaccharide with a double bond in C4–C5 at the non-reducing terminus by cell-surface hyaluronate lyase (HysA) through a β-elimination reaction^5^. Our previous reports demonstrated that the resultant unsaturated disaccharide was degraded into two constituent monosaccharides (unsaturated glucuronic acid and *N*-acetyl-d-glucosamine) by cytoplasmic unsaturated glucuronyl hydrolase (UGL)^8^, and that 4-deoxy-l-*threo*-5-hexosulose-uronic acid (α-keto acid, DHU) non-enzymatically converted from unsaturated glucuronic acid was metabolised to pyruvate and glyceraldehyde-3-phosphate by consecutive reactions of isomerase (DhuI), NADH-dependent reductase (DhuD), kinase (KdgK), and aldolase (KdgA)^9^. These enzymes (HysA, UGL, DhuI, DhuD, KdgK, and KdgA) for depolymerisation, degradation and metabolism of GAGs are coded in a GAG genetic cluster (Fig. 1A). The cluster also contains gene encoding components (enzymes IIA, B, C and D) of a putative phosphotransferase system (PTS) for the import of GAG disaccharide.

Similar GAG genetic clusters are found in the genome of various bacteria, most of which are pathogenic. Especially, the gene coding for UGL is universally included in GAG clusters, and the enzyme is unique in the degradation of unsaturated GAG disaccharides to constituent monosaccharides. On the other hand, there is a variety in the genetic organisation (e.g. import system and metabolic enzymes) in the GAG clusters. In place of PTS, we have recently identified an ATP-binding cassette transporter encoded in the GAG cluster of *Streptobacillus moniliformis* as a first importer of unsaturated GAG disaccharides to the bacterial cells^10^ (Fig. 1B). Two genes for isomerase KduI and reductase KduD responsible for pectin metabolism^11^ are also included in the GAG cluster, and KduI and KduD seem to correspond to DhuI and DhuD, respectively^9^, although the action of KduI and KduD toward GAG-derived products remains unclear.

To the best of our knowledge, no reports on the degradation of GAGs by *Lactobacillus*, a typical member of probiotics, have been published, although some strains of the genus are attached to host cells by GAG binding^4^. For GAG-targeting bacteria including probiotics, GAGs are attractive nutritionally because polysaccharides are constitutively produced on the cell surface by hosts. Thus, studies on the mechanism of degradation and metabolism of GAGs by probiotics will contribute to the development of novel prebiotics for the enhanced colonisation of beneficial bacteria in the human gut. This article deals with the degradation and metabolism of GAGs by probiotics and adhesion of GAG-degrading probiotics to human intestine cells via GAG. The predominance of *Bacteroides* in human gut microbiota was also examined through a GAG degradation analysis and metagenomics regarding the UGL gene.

## Results

### GAG degradation by human gut microbiota

**Figure 2:**
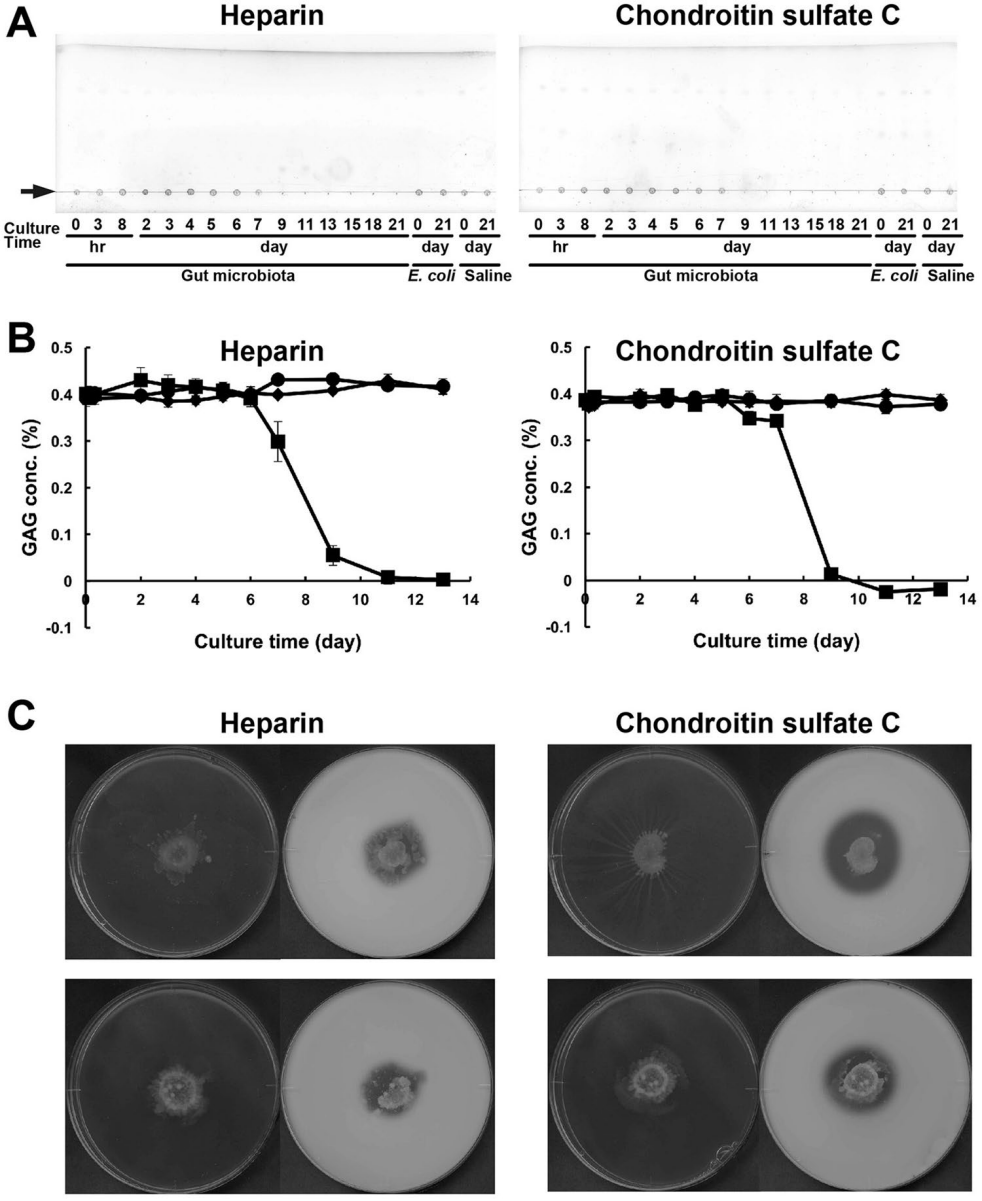
Degradation profiles of GAGs by human gut microbiota. Gut microbiota from human faeces were co-cultured for 21d in the nutrition medium containing heparin (left) and chondroitin sulfate C (right). The supernatants derived from periodically sampled culture broth were subjected to TLC (**A**) and GAG assays (**B**). Due to polysaccharides, GAGs on the TLC plates remained at the origin, indicated by an arrow (**A**). Square, round and diamond-shaped symbols represent samples from the co-culture of gut microbiota, *E*. *coli* culture and saline, respectively, in GAG degradation profiles (**B**). *E. coli* cells or the saline used as a negative control exhibited no GAG-degrading ability. After co-culture for 6d, spots at the origin corresponding to both heparin and chondroitin sulfate C as polysaccharides were found to be gradually diminished, and were completely degraded in the co-culture sample by day 9. Degradation profile by TLC (**A**) coincided with that by GAG assay (**B**). Degradation of heparin and chondroitin sulfate C by gut microbiota from Japanese man in his 50’s (upper) and woman in her 20’s (lower) was also confirmed by plate assay for halo detection (**C**). After growth of gut microbiota on GAG-containing nutrition medium plate for 7d (left), acetic acid was added on the plate for halo formation (right). The remaining GAG and BSA formed a complex as white precipitates. These profiles are not an image cropped from different parts of the same TLC or agar plate, or from different TLC or agar plates.

In this study, three GAGs, i.e. heparin, chondroitin sulfate C and hyaluronan were used as substrates for bacterial degradation (Fig. S1). GAG degradation was examined by a co-culture of human gut microbiota. Microbes included in the faeces of Japanese man in his 20’s were anaerobically co-cultured in a nutrition medium containing heparin or chondroitin sulfate C. The culture broth was periodically sampled and subjected to thin-layer chromatography (TLC) and a GAG colorimetric assay (Fig. 2). No GAG degradation was observed by *Escherichia coli* cells or in the saline used as a negative control. After co-culture for 6d, spots at the origin corresponding to both heparin and chondroitin sulfate C were found to be gradually diminished, and were completely degraded in the co-culture sample by day 9 (Fig. 2A). Similarly, GAG concentration in the culture broth decreased between days 7 and 9 (Fig. 2B). Degradation of heparin and chondroitin sulfate C by gut microbiota was also observed in the other Japanese man in his 50’s and woman in her 20’s (Fig. S2). Gut microbiota from these two Japanese formed a clear halo^12^ on the medium plate containing heparin or chondroitin sulfate C, revealing that GAG-degrading microbes were included in the human gut microbiota (Fig. 2C). The results obtained here demonstrated that GAGs were completely degraded by human gut microbiota.

### GAG-degrading bacteria isolated from human gut microbiota

**Figure 3:**
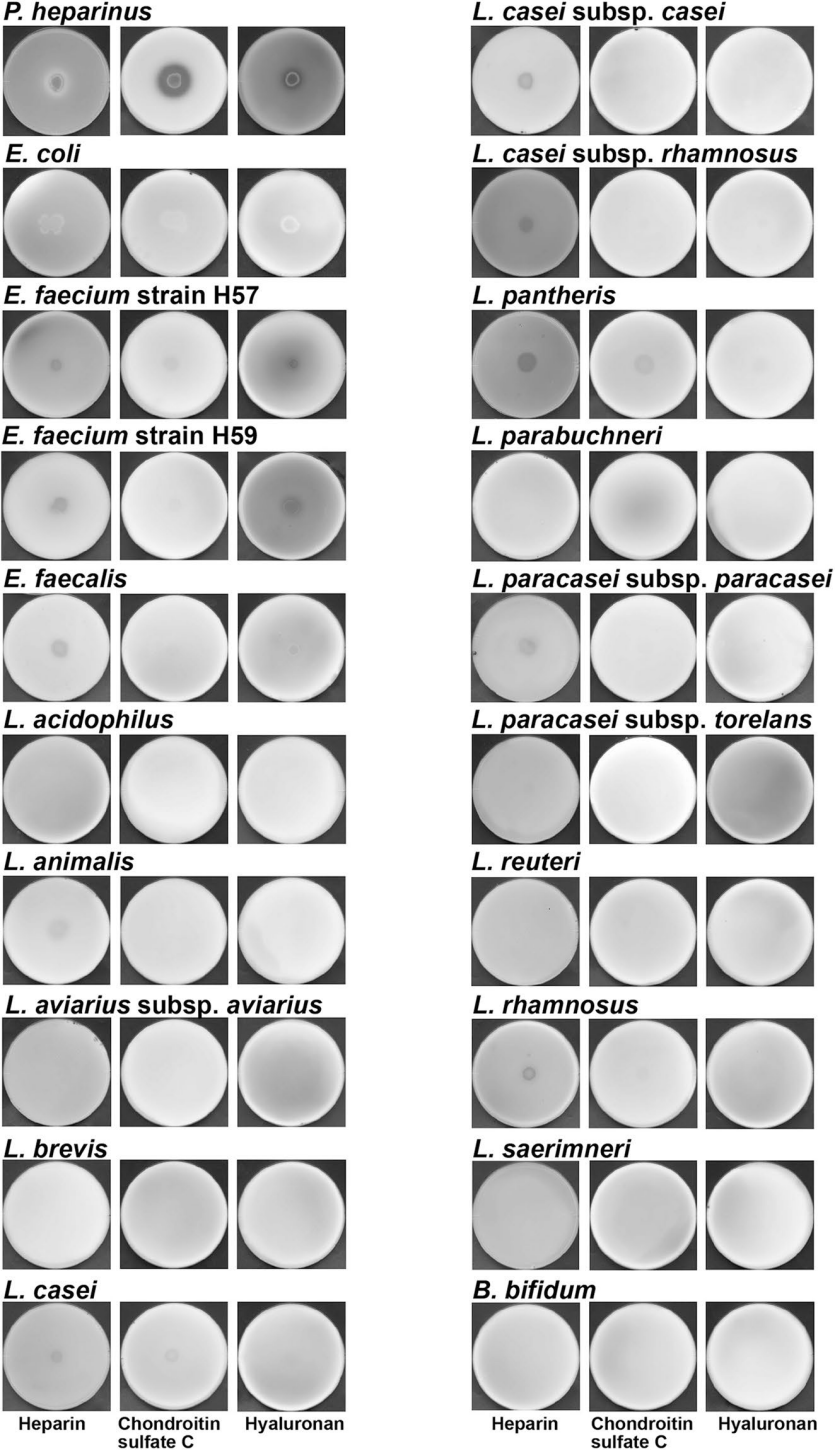
Degradation of GAGs by probiotics. Various bacterial cells, including probiotic *Enterococcus*, *Lactobacillus*, and *Bifidobacterium* species were grown at the centre of the GAG + YE0.1 + BSA plate containing heparin (left), chondroitin sulfate C (centre) or hyaluronan (right) and subjected to GAG staining with acetic acid. *P*. *heparinus* and *E*. *coli* cells were used as positive and negative controls for GAG degradation, respectively.

Two GAG-degrading bacteria termed strains H57 and H59 were isolated from human faeces by anaerobic enrichment cultivation using GAG-included plates. Degradation of GAGs (heparin, chondroitin sulfate C and hyaluronan) was further investigated by a plate assay for halo (clear zone) detection^12^ (Fig. 3). GAG-degrading *Pedobacter heparinus* used as a positive control formed halos on all the three plates containing the GAGs tested here, while no GAG degradation by *E*. *coli* used as a negative control was observed. Strain H57 and H59 cells were found to degrade heparin and hyaluronan, and a faint halo was observed on the chondroitin sulfate C plate with strain H57 cells. On the basis of the 16S rRNA sequence analysis, both strains H57 and H59 were identified as members of the probiotic *Enterococcus faecium*^13^, while this species is also known to be an opportunistic pathogen^14^.

Since strains H57 and H59 were a first example of enterococci degrading GAGs, strain H57 was subjected to the draft genome sequence analysis. The total coding length of the whole H57 genome was 2,583,114, and its reads were assembled into 108 contigs. Each contig was designed as NODE_1 to 173 in the GenBanK/EMBL/DDBJ databases (Table S1). The key enzyme UGL for GAG degradation was encoded in NODE_71, and putative genes coding for NagE (PTS, *N*-acetylglucosamine-specific EIICBA components) and metabolic enzymes (KduI, KduD, KdgK, and KdgA) of α-keto acid (DHU) were included in NODE_35 and 92, respectively (Fig. 1E).

### Degradation and metabolism of GAGs by probiotics

Distinct from pathogens such as streptococci and clostridia, the probiotic strains H57 and H59 were found to degrade GAGs and some typical probiotics including *Enterococcus*, *Lactobacillus*, and *Bifidobacterium* (Table 1) were also subjected to the plate assay for GAG degradation (Fig. 3). Heparin was degraded by *Enterococcus faecalis*, *Lactobacillus animalis*, *Lactobacillus casei*, *Lactobacillus casei* subsp. *casei*, *Lactobacillus casei* subsp. *rhamnosus*, *Lactobacillus pantheris*, *Lactobacillus paracasei* subsp. *paracasei* and *Lactobacillus rhamnosus*. In addition to heparin, *L*. *casei* and *L*. *pantheris* seemed to slightly degrade chondroitin sulfate C. Degradation of any GAGs was hardly detected by the following probiotics: *Lactobacillus acidophilus*, *Lactobacillus aviarius* subsp. *aviarius*, *Lactobacillus brevis*, *Lactobacillus parabuchneri*, *Lactobacillus paracasei* subsp. *torelans*, *Lactobacillus reuteri*, *Lactobacillus saerimneri* and *Bifidobacterium bifidum*.

**Table 1:** List of bacteria used in this study and their preculture condition.

Bacterial species	Growth condition		Medium
	Temp. (°C)	Oxygen	
*Bacteroides clarus* JCM 16067	30	Anaerobic	GAM
*Bacteroides dorei* JCM 13471	37	Anaerobic	GAM
*Bacteroides helcogenes* JCM 6297	37	Anaerobic	GAM
*Bacteroides intestinalis* JCM 13265	37	Anaerobic	GAM
*Bacteroides ovatus* JCM 5824	37	Anaerobic	GAM
*Bacteroides paurosaccharoliticus* JCM 15092	30	Anaerobic	GAM
*Bacteroides salanitronis* JCM 13657	37	Anaerobic	GAM
*Bacteroides stercoris* JCM 9496	37	Anaerobic	GAM
*Bacteroides thetaiotaomicron* JCM 5827	37	Anaerobic	GAM
*Bacteroides uniformis* JCM 5828	37	Anaerobic	GAM
*Bacteroides vulgatus* NBRC 14291	37	Anaerobic	GAM
*Bifidobacterium bifidum* NBRC 100015	37	Anaerobic	MRS
*Enterococcus faecalis* NBRC 100480	37	Anaerobic	MRS
*Enterococcus faecium* strain H57	37	Anaerobic	MRS
*Enterococcus faecium* strain H59	37	Anaerobic	MRS
*Escherichia coli* K-12 MG1655	30	Anaerobic	LB
*Lactobacillus acidophilus* NBRC 13951	37	Anaerobic	MRS
*Lactobacillus animalis* NBRC 15882	37	Anaerobic	MRS
*Lactobacillus aviarius* subsp. *aviarius* NBRC 102162	37	Anaerobic	MRS
*Lactobacillus brevis* NBRC 107147	30	Anaerobic	MRS
*Lactobacillus casei* NBRC 15883	37	Anaerobic	MRS
*Lactobacillus casei* subsp. *casei* ATCC 334	37	Anaerobic	MRS
*Lactobacillus casei* subsp. *rhamnosus* ATCC 8530	37	Anaerobic	MRS
*Lactobacillus pantheris* NBRC 106106	37	Anaerobic	MRS
*Lactobacillus parabuchneri* NBRC 107865	30	Anaerobic	MRS
*Lactobacillus paracasei* subsp. *paracasei* NBRC 15889	37	Anaerobic	MRS
*Lactobacillus paracasei* subsp. *tolerans* NBRC 15906	37	Anaerobic	MRS
*Lactobacillus reuteri* NBRC 15892	37	Anaerobic	MRS
*Lactobacillus rhamnosus* NBRC 3425	37	Non-aerobic	MRS
*Lactobacillus saerimneri* NBRC 107826	37	Anaerobic	MRS
*Pedobacter heparinus* NBRC 12017	30	Non-aerobic	802^*a*^

^*a*^The medium 802 consists of 1% hipolypeptone, 0.2% yeast extract and 0.1% MgSO_4_·7H_2_O.

**Figure 4:**
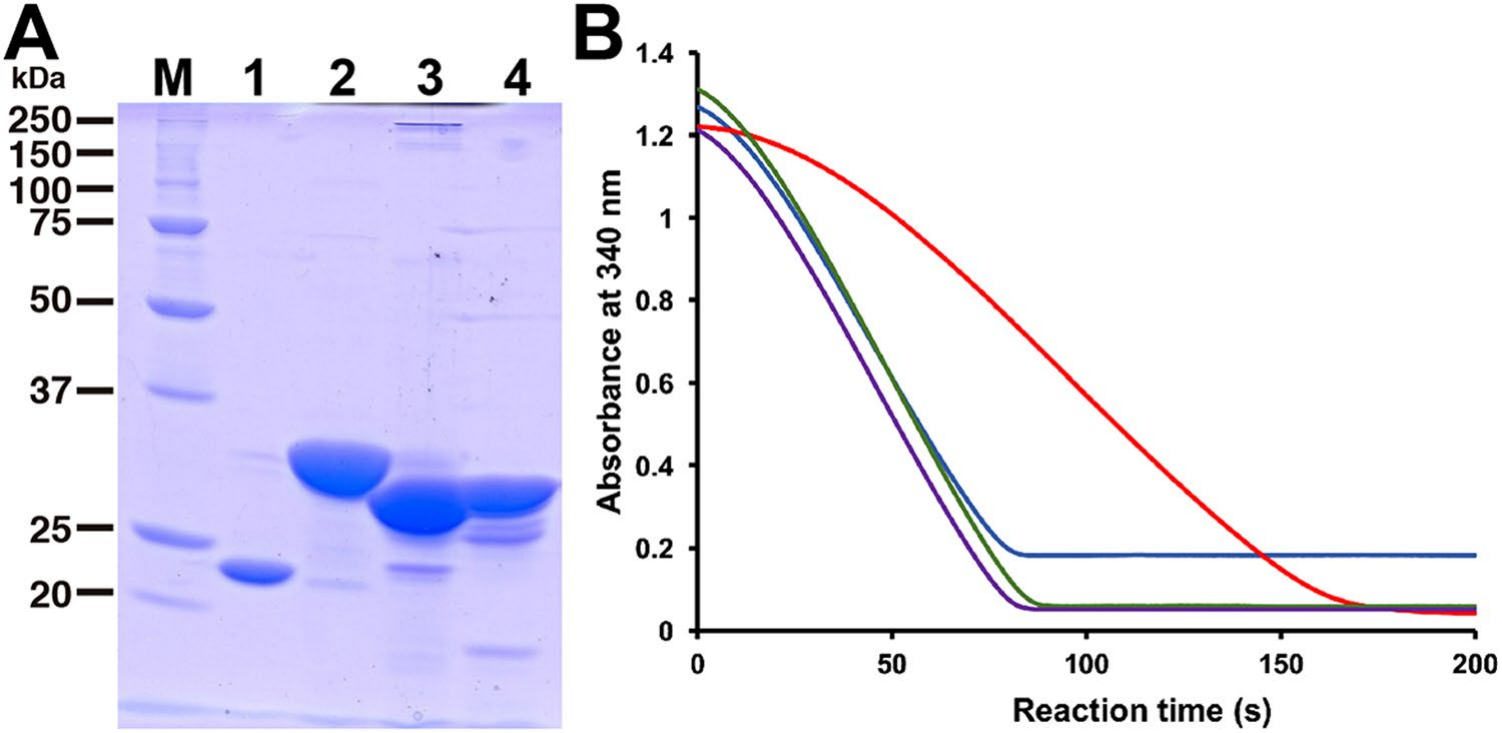
KduI and KduD involved in GAG metabolism. (**A**) SDS-PAGE profile of purified DhuI, DhuD, KduI and KduD. Lane M, molecular weight markers; lane 1, DhuI; lane 2, DhuD; lane 3, KduI; and lane 4, KduD. This profile is not an image cropped from different parts of the same gel or from different gels. (**B**) NADH oxidation (decrease in absorbance at 340 nm) through a successive reaction of isomerase (DhuI or KduI) and reductase (DhuD or KduD) in the presence of the substrate (DHU). The substrate was prepared from unsaturated chondroitin disaccharide with a sulfate group at C6 position through the reaction of UGL. Blue, the combination of DhuI and DhuD; purple, the combination of DhuI and KduD; green, the combination of KduI and DhuD; and red, the combination of KduI and KduD.

Among GAG-degrading lactobacilli, some strains classified to *L*. *casei*, *L*. *paracasei* and *L*. *rhamnosus* were found to contain the GAG genetic cluster in the bacterial genome (Fig. 1C,D). The recombinant *L*. *rhamnosus* HepC annotated as heparinase II/III family protein encoded in the bacterial GAG genetic cluster seemed to degrade heparin through a β-elimination reaction (Iwase *et al*. Abstract paper presented at Regular Meeting of Kansai Branch of the Japan Society for Bioscience, Biotechnology, and Agrochemistry, held in Kyoto, Japan on February 3, 2018). Two genes coding for KduI and KduD were also included in the *Lactobacillus* cluster, although both are generally located in the genome segment of pectin-degrading bacteria^11^. KduI and KduD were first identified to be isomerase and reductase, respectively, for the metabolism of unsaturated galacturonic acid generated from pectin^15,16^. On the other hand, there is no experimental evidence for involvement of these KduI and KduD in metabolism of GAGs. Due to high sequence identity among *Lactobacillus* KduIs or KduDs, two recombinant KduI and KduD of *L*. *rhamnosus* were expressed as typical *Lactobacillus* proteins in *E*. *coli* cells and purified to homogeneity (Fig. 4A).

α-Keto acid DHU non-enzymatically produced from unsaturated uronic acids from GAGs is converted to 3-deoxy-d-*glycero*-2,5-hexodiulosonic acid (DK-II) by DhuI^9^. The resultant product is reduced to 2-keto-3-deoxy-d-gluconic acid (KDG) by NADH-dependent DhuD. Similar to DhuI and DhuD, KduI and KduD were found to be crucial for the metabolism of DHU. A combination assay using isomerase (KduI or DhuI) and reductase (KduD or DhuD) indicated that KduI and KduD function as 4-deoxy-l-*threo*-5-hexosulose-uronate ketol-isomerase and 2-keto-3-deoxy-d-gluconate dehydrogenase, respectively (Fig. 4B).

### GAG-degrading probiotics attached to intestinal Caco-2 cells

**Figure 5:**
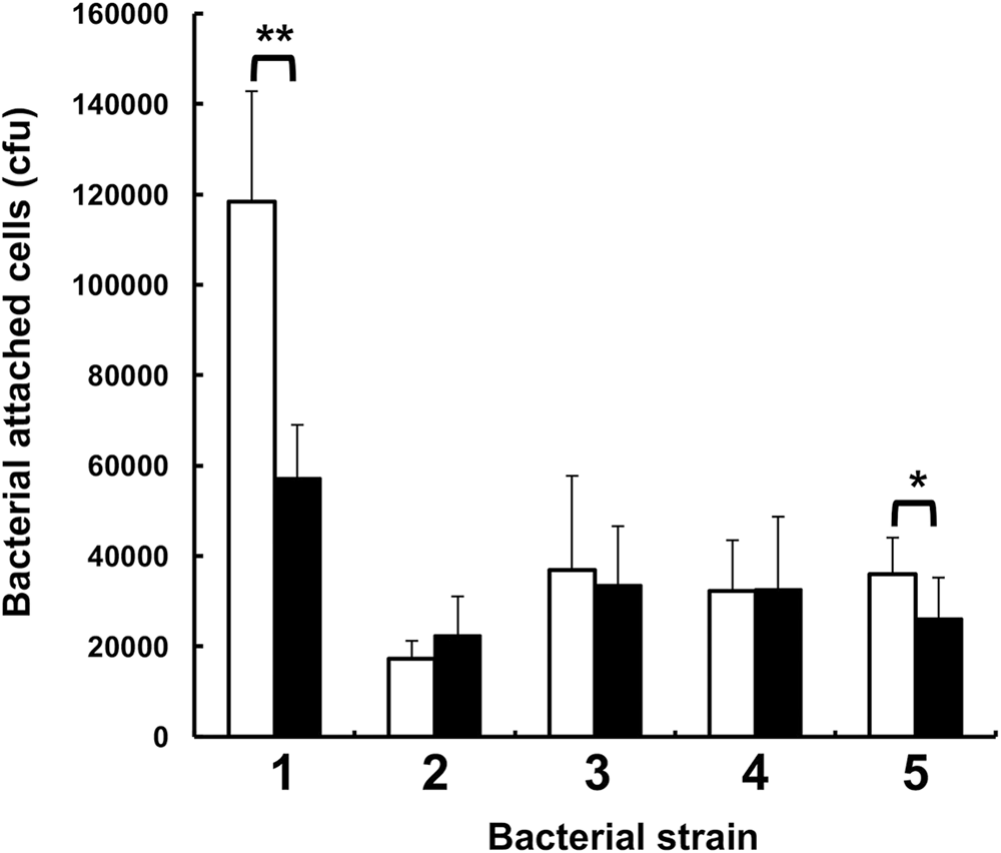
Bacterial attachment to Caco-2 cells. Bacterial cells attached to human intestinal cells were measured in each well in the presence (closed bar) and absence (open bar) of heparin. Strain 1, *L*. *rhamnosus*; strain 2, *L*. *casei* subsp. *rhamnosus*; strain 3, *L*. *casei*; strain 4, *L*. *animalis*; and strain 5, *E*. *faecium* strain H57. The adhesion of *L*. *rhamnosus* and *E*. *faecium* strain H57 was significantly decreased in the presence of heparin. Significant difference was statistically determined by Student’s t-test (**p < 0.01; *p < 0.05). The results are presented as means ± SD. Experiments were conducted independently in triplicate and cfu in each experiment was determined by three measurements.

Although some lactobacilli and enterococci have been reported to show an adherence to Caco-2 cells^17,18^, we investigated the ability of GAG-degrading probiotics to attach to intestinal cells. Five probiotic heparin-degrading *E*. *faecium* strain H57, *L*. *animalis*, *L*. *casei*, *L*. *casei* subsp. *rhamnosus* and *L*. *rhamnosus* were subjected to the competition binding assay (Fig. 5). All the probiotics showed an ability to attach to intestinal cells, although various adherence levels were observed, depending on the bacterial species. The adhesion of *E*. *faecium* strain H57 and *L*. *rhamnosus* to Caco-2 cells was significantly decreased by the addition of heparin, while in the case of the other three probiotics, there was no significant difference in their adhesion to intestinal cells between bacterial cells in the presence and absence of heparin.

### Frequent detection of *Bacteroides* UGL gene in human gut microbiota

**Figure 6:**
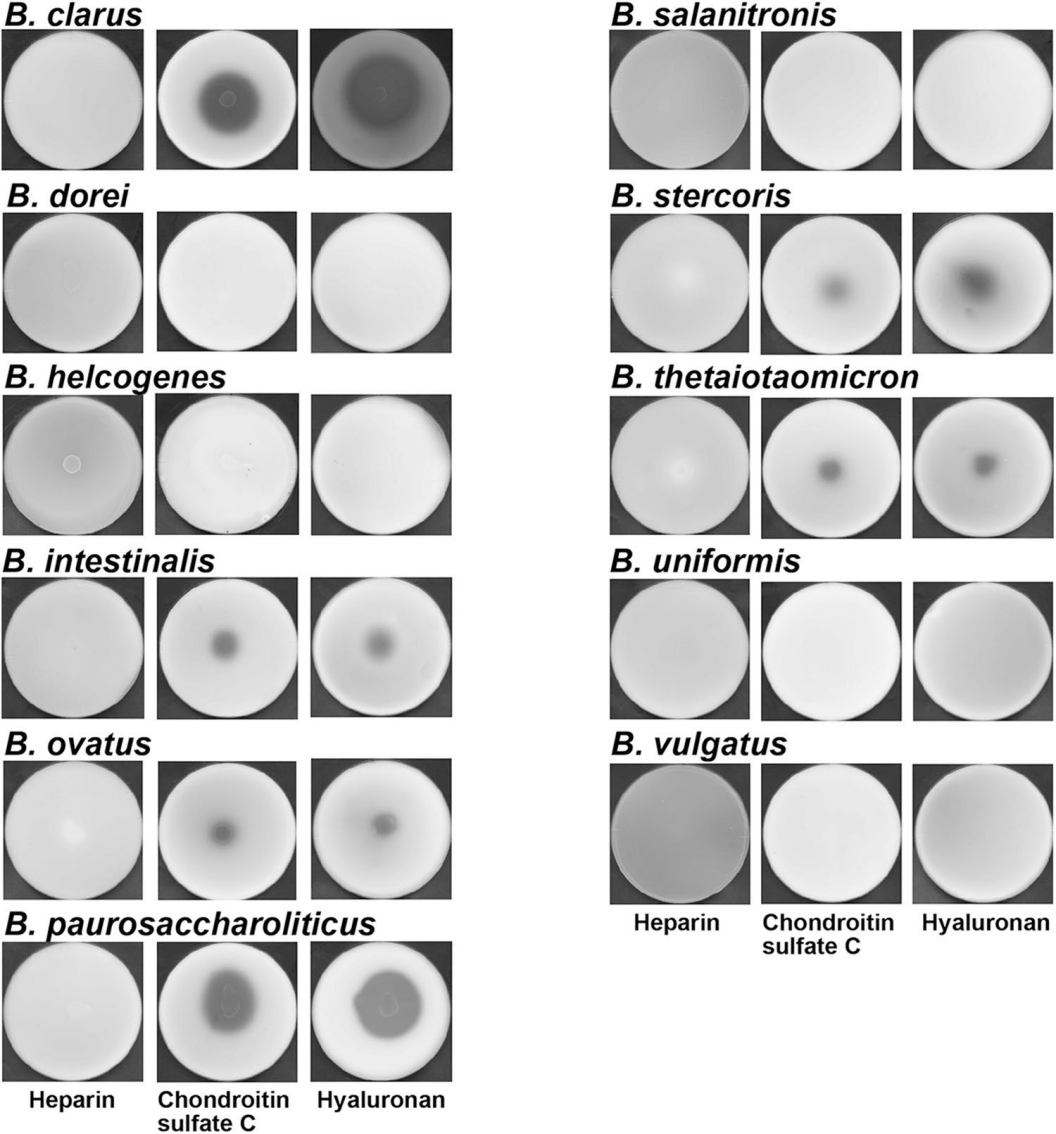
Degradation of GAGs by *Bacteroides* species. Bacterial cells were grown at the centre of the GAG + YE0.1 + BSA plate containing heparin (left), chondroitin sulfate C (centre), or hyaluronan (right) and subjected to GAG staining with acetic acid.

Distinct from lactobacilli probiotics, which are a minor bacterial component in human gut microbiota, several kinds of *Bacteroides* species predominant in the microbiota have been reported to degrade GAGs^19–23^. Recently, some *Bacteroides* species have been considered to be next generation probiotics beneficial to human health^24,25^, although the genus has long been known to be an opportunistic pathogen. In order to investigate the involvement of *Bacteroides* GAG-degrading ability in their predominance in the microbiota, 11 *Bacteroides* species, including the known GAG-degrading *Bacteroides intestinalis*, *Bacteroides ovatus*, *Bacteroides stercoris* and *Bacteroides thethaiotaomicron* were subjected to the plate assay for GAG degradation (Fig. 6). In addition to the above four species, two species, i.e. *Bacteroides clarus* and *Bacteroides paurosaccharoliticus*, were found to degrade chondroitin sulfate C and hyauronan, while heparin was inert as a substrate for degradation by any *Bacteroides* species. Therefore, six *Bacteroides* species tested here were demonstrated to have a capability to degrade GAGs.

Based on the genome sequence of nine *Bacteroides* species so far determined, the UGL gene crucial for GAG degradation is found to be included in all of the *Bacteroides* genome. Thus, detection of the *Bacteroides* UGL gene was attempted by polymerase chain reaction (PCR) amplification through a metagenomic analysis using two Japanese faeces samples. According to the nucleotide sequences of five *Bacteroides* UGL genes, two specific forward and reverse primers for the *Bacteroides* UGL gene were designed, as follows: 5′-ATBGAYAAYATGATSAAVCTSGA-3′ and 5′-GCYTGHCCRCGWGMCCA-3′ (Table S2). A gene fragment with a size of about 230 bp was predicted to be amplified from the genome of *Bacteroides* species through PCR using these primers. Two DNA samples were extracted from faecal samples obtained from two men in their 20’s and 40’s. Real Time (RT)-PCR toward two DNA samples in addition to genomic DNA of *Bacteroides vulgatus* as a positive control indicated that 230 bp DNA fragments were amplified from every DNA sample (Fig. S3). Based on the nucleotide sequences of PCR products, both the amplified DNA fragments from the two DNA samples were identified to be a part of the *Bacteroides* UGL gene. In order to determine the frequency of the *Bacteroides* UGL gene in total gut microbiota, copy numbers of the 16S rRNA and *Bacteroides* UGL genes were estimated from RT-PCR analysis. In the case of the man in his 40’s, copy numbers of the 16S rRNA and *Bacteroides* UGL genes per 1 g of faeces were 5.60 × 10^10^ and 7.40 × 10^8^, respectively. Similarly, copy numbers of the 16S rRNA and *Bacteroides* UGL genes per 1 g of faeces from the man in his 20’s were 2.07 × 10^11^ and 6.09 × 10^9^, respectively. In this RT-PCR analysis, PCR efficiency and the correlation coefficient of the 16S rRNA gene were determined to be 0.95 and 0.999, respectively, and those of the *Bacteroides* UGL gene were 0.79 and 0.999, respectively.

## Discussion

In the present study, we found GAG-degrading probiotics in human gut microbiota and identified their ability to show an adherence to intestinal cells through GAG for the first time. Retardant degradation of GAGs (heparin and chondroitin sulfate C) was observed by the co-culture of human gut microbiota in the nutrition medium (Figs 2 and S2), suggesting that other nutrition factors such as yeast extract and/or tryptone rather than GAGs are readily utilised, but after their consumption, GAGs were available for the microbiota and they were finally completely degraded. Therefore, human gut microbiota are considered to usually utilise food-derived nutrition factors, and subsequently GAGs produced by hosts also become targets for degradation by some genera in the microbiota. Unsaturated disaccharides have been reported to be accumulated as an end-product through reactions by degradation of chondroitin sulfates by gut microbiota in the human Chinese populations^7^. On the other hand, in the case of GAG degradation by Japanese gut microbiota, no depolymerisation products such as unsaturated GAG disaccharides were observed after prolonged reaction on the TLC plate (Fig. 2A and Fig. S2), suggesting that after depolymerisation of GAGs by polysaccharide lyases, the resultant disaccharides were finally incorporated to the cytoplasm of the lyase producers and/or other bacteria.

Although some enterococci have been demonstrated to show an adherence to intestinal cells through heparin and/or heparan sulfate^26^, probiotic *E*. *faecium* strains H57 and H59 were isolated as the first GAG-degrading enterococci from human gut microbiota. Interestingly, no homologous genes coding for GAG lyases such as heparin lyases I, II, and III^27,28^ were observed in the genome of *E*. *faecium* strain H57. In comparison with the predominant genus *Bacteroides* in human gut microbiota (Fig. 6), enterococci showed less ability to degrade GAGs (Fig. 3), suggesting that enterococci utilise unsaturated GAG disaccharides produced by other bacteria. In fact, homologous proteins and enzymes essential for the import, degradation and metabolism of unsaturated GAG disaccharides are encoded in the genome of strain H57 (Fig. 1E).

**Figure 7:**
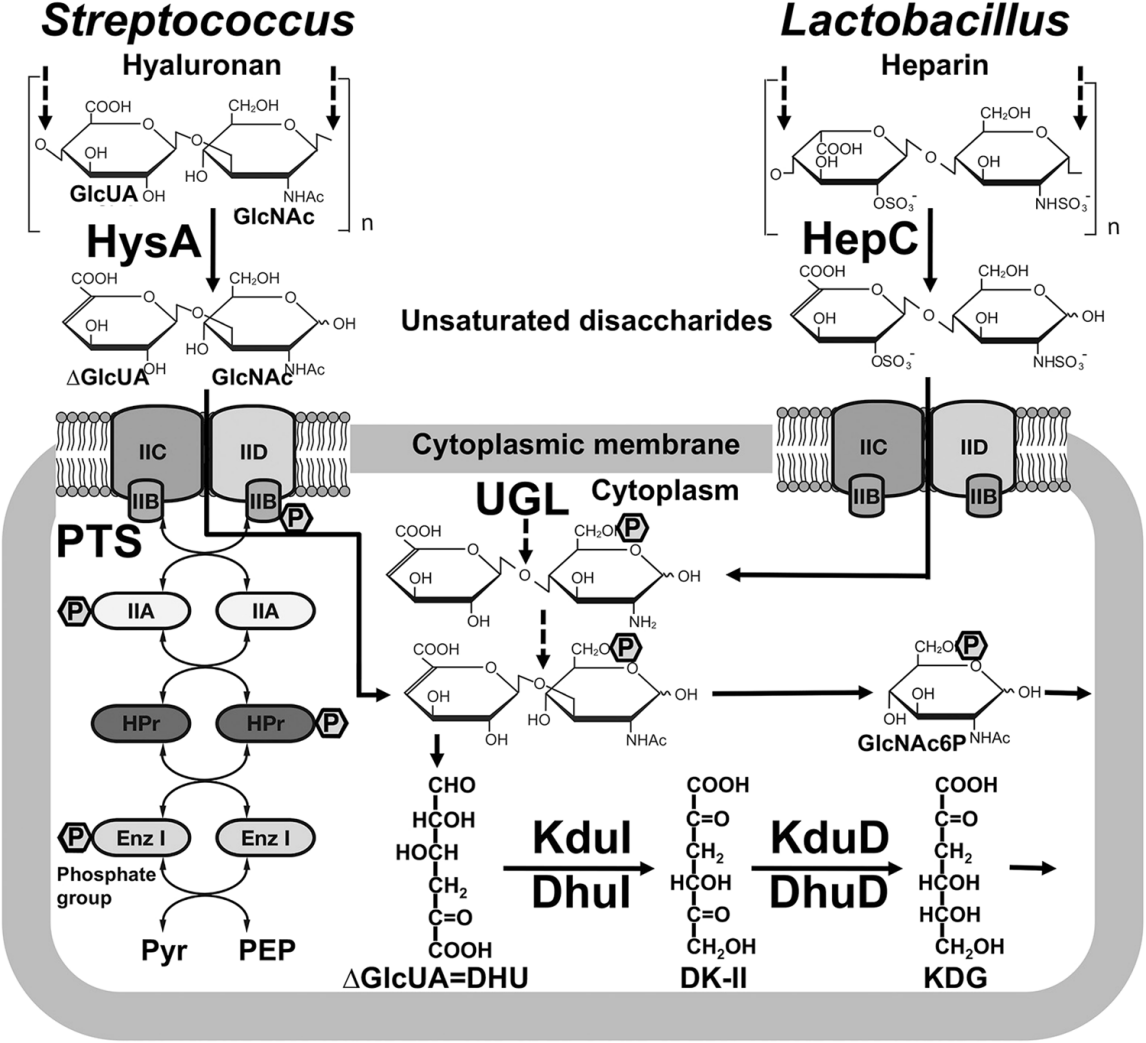
Bacterial system for the degradation and metabolism of GAGs. *Streptococcus* and *Lactobacillus* systems for the degradation and metabolism of GAGs. GAGs such as hyaluronan (left) and heparin (right) were depolymerised to unsaturated disaccharides by cell-surface or extracellular polysaccharide lyases (PL8 family hyaluronate lyase HysA and PL12 family heparin lyase HepC). The resultant disaccharides were imported to the cytoplasm by putative PTS and degraded to constituent monosaccharides (unsaturated uronic acid and amino sugar) by cytoplasmic UGL before or after desulfation by sulfatase. DHU non-enzymatically produced from unsaturated uronic acid was converted to KDG via an intermediate DK-II through a sequential reaction by isomerase (DhuI or KduI) and NADH-dependent reductase (DhuD or KduD).

Molecular systems of depolymerisation, import, degradation and metabolism of GAGs have functionally and structurally been characterised in some pathogenic bacteria^5,6,8–10^ (Fig. 7, left). Genes coding for enzymes and proteins constituting the molecular systems are assembled to a cluster in some bacteria such as *Streptococcus*, *Clostridium* and *Streptobacillus* (Fig. 1). Other than such pathogenic bacteria, some probiotic lactobacilli are found to contain a similar GAG genetic cluster in their genomes. In fact, among GAG-degrading *Lactobacillus* species, i.e. *L*. *animalis*, *L*. *casei*, *L*. *pantheris*, *L*. *paracasei* and *L*. *rhamnosus* shown here, *L*. *casei*, *L*. *paracasei* and *L*. *rhamnosus* included the GAG genetic cluster, although no genome sequence of *L*. *animalis* and *L*. *pantheris* is available on the KEGG database (http://www.genome.jp/kegg/catalog/org_list.html).

As shown in Fig. 1C,D, the GAG cluster of *Lactobacillus* encodes KduI and KduD in place of streptococcal isomerase DhuI and reductase DhuD. Due to a lack of the hydroxyl group at C4 position, unsaturated glucuronic and iduronic acids generated from GAGs are identical to unsaturated galacturonic acid^9^, suggesting that the *Lactobacillus* KduI and KduD correspond to streptococcal GAG-metabolising DhuI and DhuD, respectively, although there is little sequence similarity between KduI and DhuI and between KduD and DhuD. Enzyme characterisation of the recombinant *Lactobacillus* KduI and KduD indicated that 4-deoxy-l-*threo*-5-hexosulose-uronate ketol-isomerase KduI catalyses the isomerase reaction from DHU to DK-II, which is further reduced to KDG by NADH-dependent reductase KduD. This is the first report to identify that KduI and KduD are crucial for GAG metabolism, and that these two metabolic enzymes together with KdgK and KdgA are also encoded in the genome of *E*. *faecium* strain H57 isolated as GAG-degrading bacterium from human gut microbiota (Fig. 1E). Therefore, based on the streptococcal system of action toward hyaluronan, the corresponding *Lactobacillus* model for heparin degradation was postulated in Fig. 7, right. Heparin is depolymerised to unsaturated disaccharides by heparin lyase HepC categorised to polysaccharide lyase family 12 on the basis of the primary structure. The resultant unsaturated heparin disaccharide is incorporated into cytoplasm through phosphorylation by PTS composed of EII subunits A, B, C and D. The cytoplasmic enzyme UGL degraded the unsaturated heparin disaccharide to unsaturated uronic acid and amino sugar. The resultant unsaturated uronic acid is non-enzymatically converted to DHU, which is further metabolised to pyruvate and glyceraldehyde-3-phosphate through successive reactions of isomerase KduI, NADH-dependent reductase KduD, kinase KdgK and aldolase KdgA.

The attachment of two heparin-degrading probiotics *L*. *rhamnosus* and *E*. *faecium* strain H57 to Caco-2 cells was significantly inhibited by heparin (Fig. 5), indicating that the two bacterial cells showed an adherence to the host intestinal cells through heparin, although other strains of these bacteria have been demonstrated to attach to the host cells^3,4^. On the other hand, little effect of heparin on the attachment of heparin-degrading *L*. *animalis*, *L*. *casei* and *L*. *casei* subsp. *rhamnosus* cells to the intestinal cells was observed, suggesting that these lactobacilli showed less ability to bind to the extracellular matrix heparin of Caco-2 cells. Therefore, further experiments are required to clarify the involvement of the bacterial GAG-degrading ability in the attachment to host cells. At least, GAG-degrading bacteria are beneficial for their residence and/or colonisation in host tissues because GAGs are constitutively produced on the cell surface by hosts.

Although several *Bacteroides* species in intestinal microflora are known to degrade GAGs^19–23^, two species, i.e. *B*. *clarus* and *B*. *paurosaccharolyticus*, were freshly demonstrated to degrade chondroitin sulfate C and hyaluronan (Fig. 6). Six *Bacteroides* species tested here degraded GAGs, suggesting that degradation of GAGs is well conserved in *Bacteroides* species. This was supported by metagenomics through amplification of the *Bacteroides* UGL gene, which is crucial for the degradation of unsaturated GAG disaccharides. Copy numbers of 16S rRNA genes are generally known to be 1 to 15 in the genome of bacterial cells^29^. If copy numbers of 16S rRNA and *Bacteroides* UGL genes in each bacterial cell were estimated to be 8 and 1, respectively, the ratio of bacteria having the *Bacteroides* UGL gene in total gut microbiota was determined to be 23.5% and 10.5% in the men in their 20’s and 40’s, respectively. A potent GAG degradation ability and frequent detection of the *Bacteroides* UGL gene in human gut microbiota may contribute to the predominance of *Bacteroides* species in the microbiota due to attractive nutrients (GAGs) from hosts.

In conclusion, some typical and next generation probiotics including *Lactobacillus*, *Enterococcus* and *Bacteroides* in human gut microbiota, can degrade GAGs. Genes coding for the GAGs-degrading enzyme were frequently detected from human gut microbiota, suggesting that the bacterial GAG-degrading ability may be involved in their predominance in gut microbiota.

## Materials and Methods

### Materials

Sodium heparin (heparin) was purchased from Nacalai Tesque. Chondroitin sulfate C sodium salt (chondroitin sulfate C) was purchased from Wako Pure Chemical Industries. Hyaluronic acid sodium salt (hyaluronan) was purchased from Sigma-Aldrich. Generally, heparin consists of uronic (glucuronic or iduronic) acid and glucosamine, both of which are linked by the 1,4-glycoside bond and are frequently sulfated and *N*-acetylated. Chondroitin sulfate C has a repeating disaccharide with the 1,3-glycosidic bond that is composed of glucuronic acid and *N*-acetyl-d-galactosamine with a sulfate group at the C6 position. Hyaluronan with no sulfate group is composed of glucuronic acid and *N*-acetyl-d-glucosamine, and shows a similar glycoside bond mode to chondroitin sulfate C. Nutrition-rich media, MRS and GAM, were purchased from BD Biosciences and the Nissui Pharmaceutical Co., respectively. Oligonucleotides were synthesised by Hokkaido System Science (Table S2). DNA-modifying enzymes were purchased from Toyobo. All other analytical grade chemicals used in this study were commercially available. Faeces were kind gifts from three Japanese volunteers in their 20’s, 40’s and 50’s. Informed consent was obtained from all subjects.

### Bacterial strains and growth conditions

A list of bacteria used in this work is represented in Table 1. *B*. *vulgatus* NBRC 14291, *B*. *bifidum* NBRC 100015, *E*. *faecalis* NBRC 100480, *L*. *acidophilus* NBRC 13951, *L*. *animalis* NBRC 15882, *L*. *aviarius* subsp. *aviarius* NBRC 102162, *L*. *brevis* NBRC 107147, *L*. *casei* NBRC 15883, *L*. *pantheris* NBRC 106106, *L*. *parabuchneri* NBRC 107865, *L*. *paracasei* subsp. *paracasei* NBRC 15889, *L*. *paracasei* subsp. *tolerans* NBRC 15906, *L*. *reuteri* NBRC 15892, *L*. *rhamnosus* NBRC 3425, *L*. *saerimneri* NBRC 107826 and *P*. *heparinus* NBRC 12017 were purchased from the National Institute of Technology and Evaluation, Japan. *L*. *casei* subsp. *casei* ATCC 334 and *L*. *casei* subsp. *rhamnosus* ATCC 8530 were from the American Type Culture Collection. *B*. *clarus* JCM 16067, *B*. *dorei* JCM 13471, *B*. *helcogenes* JCM 6297, *B*. *intestinalis* JCM 13265, *B*. *ovatus* JCM 5824, *B*. *paurosaccharoliticus* JCM 15092, *B*. *salanitronis* JCM 13657, *B*. *stercoris* JCM 9496, *B*. *thetaiotaomicron* JCM 5827 and *B*. *uniformis* JCM 5828 were from the Japan Collection of Microorganisms. Unless otherwise stated, the standard condition for bacterial growth in nutrition media is also described in Table 1.

### Co-culture of human gut microbiota

A medium for the co-culture of human gut microbiota [0.4% (w/v) GAG (heparin or chondroitin sulfate C), 0.45% yeast extract, 0.3% tryptone, 0.3% peptone, 0.08% l-cysteine hydrochloride, 0.45% NaCl, 0.25% KCl, 0.045% MgCl_2_·6H_2_O, 0.02% CaCl_2_·2H_2_O and 0.04% KH_2_PO_4_] was prepared according to the previous report with a slight modification^30^. A small portion of human faeces was resuspended in 1.5 ml saline (0.15 M NaCl) and 10 µl of the faecal suspension was added to 10 ml of the co-culture medium. Gut microbiota from faeces of two men in their 20’s and 50’s and a woman in her 20’s were anaerobically cultivated at 37 °C. In place of the faecal suspension, saline or a cell suspension of *E*. *coli* used as a negative control was also anaerobically incubated at 37 °C. A portion of the culture broth was periodically extracted for determination of GAG concentration. After centrifugation at 9,700 × g for 5 min at room temperature, the supernatant was stored at −20 °C until ready for use.

### Isolation of GAG-degrading bacteria from human gut microbiota

To isolate GAG-degrading bacteria from human gut microbiota, three kinds of media were used, as follows: (i) GAG minimal plate [0.2% GAG (heparin or chondroitin sulfate C) as a sole carbon and nitrogen source, 0.1% Na_2_HPO_4_, 0.1% KH_2_PO_4_, 0.01% MgSO_4_·7H_2_O and 1.5% agar], (ii) GAG + YE0.1 plate [0.2% GAG (heparin or chondroitin sulfate C), 0.1% yeast extract, 0.1% Na_2_HPO_4_, 0.1% KH_2_PO_4_, 0.01% MgSO_4_·7H_2_O and 1.5% agar], and (iii) GAG + YE0.5 plate [0.2% GAG (heparin or chondroitin sulfate C), 0.5% yeast extract, 0.1% Na_2_HPO_4_, 0.1% KH_2_PO_4_, 0.01% MgSO_4_·7H_2_O and 1.5% agar]. For maintenance and/or preculture, the bacterial cells were grown in nutrition-rich media such as LB plate (1% tryptone, 0.5% yeast extract, 1% NaCl and 1.5% agar) and/or MRS plate. The above-described human gut microbiota were anaerobically or non-aerobically cultured at 37 °C in each plate. Few colonies formed on the GAG minimal and GAG + YE0.1 plates. Some colonies were anaerobically grown on the GAG + YE0.5 plate and these were picked up with a sterilised tooth pick for streak culture on the GAG + YE0.1 plate to separate single colonies. The oxygen conditions of further cultivated colonies were consistent with those of the initial isolation cultivation. Finally, two strains H57 and H59 were isolated from the heparin + YE0.5 plate as a heparin-degrading microbe through the plate assay for GAG degradation after cultivation on the GAG + YE0.1 + bovine serum albumin (BSA) plate for 4 weeks. Strain H57 and H59 cells were identified through a 16S rRNA sequence analysis using the standard 27f and 1492r primers (Table S2).

### Rapid plate method for the detection of GAG-degrading microbes

GAGs are known to form a complex with BSA and the complex is converted to white precipitates in the presence of acetic acid^12^. After inoculation and cultivation of bacterial cells at the centre of medium plates containing BSA and each GAG, acetic acid was poured on the plates to form white precipitates in complex with GAGs and BSA. In the case of GAG-degrading microbes grown on GAG and BSA-included medium plates, a clear zone (halo) appeared around the microbial cells after the addition of acetic acid, because no precipitation by the degraded GAG and BSA occurred. Microbial cells from human gut microbiota or standard bacterial cells such as *Lactobacillus* and *Bacteroides* species were precultured in nutrition-rich medium (Table 1) and the optical density at 600 nm (OD_600_) of the preculture broth was measured. Microbial cells were centrifuged at 9,700 × g for 5 min at room temperature, washed with 1 ml of 0.15 M NaCl and resuspended in the saline. The volume (X µl) of the saline to the prepare cell suspension was calculated based on the following formula: X = 200 × OD_600_. The resultant cell suspension was subjected to the plate assay for GAG degradation. A portion (10 µl) of the cell suspension was spotted at the centre of the BSA-included GAG + YE0.1 plate, and cultured at 30 °C or 37 °C for 7d. After cell growth on the plate, 1 ml of 2 M acetic acid was added to form a complex with the remaining GAG and BSA as white precipitates.

### Adhesion assay

Caco-2 cells were purchased from the Riken Bio Resource Center. The intestinal cells were seeded to 12-well plates and grown at 37 °C under a 5% CO_2_ atmosphere in Dulbecco’s Modified Eagle’s minimal essential medium (DMEM) containing 10% fetal bovine serum (Biological Industries) and 1% of a non-essential amino acid. For the bacterial adhesion assay, Caco-2 cells were cultured for 21d after seeding and the number of cells was adjusted to 4.0 × 10^5^ to 6.0 × 10^5^ per each well. After confluent, medium was changed every other day. Bacterial cell suspension was prepared in a similar way as mentioned in the section on the rapid plate method, with some modifications. In the adhesion assay, 1 ml of the preculture was used to prepare a bacterial cell suspension in DMEM in the place of 0.15 M NaCl. The volume (Y µl) of DMEM was calculated based on the following formula: Y = 4,000 × OD_600_. The adhesion of bacterial cells to the monolayer of Caco-2 cells was investigated according to previous reports, with some modifications^31,32^. Each well was washed twice with 1 ml of phosphate-buffered saline (PBS) and 0.5 ml of DMEM or 0.5 ml of 30 µg/ml heparin in DMEM was added to each well. The bacterial cell suspension (0.5 ml) was further added to each well and incubated at 37 °C under 5% CO_2_ atmosphere for 1 h to adhere to Caco-2 cells. Caco-2 cells incubated with bacterial cells were washed three times with 1 ml PBS to exclude non-specifically bound bacterial cells. After washing, 1 ml of 0.25% (w/v) triton X-100 in PBS was added to each well and incubated at 37 °C under 5% CO_2_ atmosphere for 1 h to lyse Caco-2 cells. The bacterial cells showed a resistance to the treatment with triton X-100. The cell lysate containing bacterial cells was spread on the MRS plate after serial diluting. Colony-forming units (cfu) were counted as numbers of bacterial cells attached to Caco-2 cells after cultivation at 37 °C for 1–2d. The number of bacterial cells attached to Caco-2 cells were presented as an average ± standard deviation (SD). A significant difference in bound bacterial cells between the presence and absence of heparin was determined by a Student’s t-test.

### Metagenomics regarding the *Bacteroides* UGL gene in human gut microbiota

Specific primers of the *Bacteroides* UGL gene were designed based on the nucleotide sequences of genes coding for five *Bacteroides* UGLs (Bache_1615 of *B. helcogenes* P 36–108, BT3348 of *B. thetaiotaomicron* VPI-5482, BXY_37650 of *Bacteroides xylanisolvens* XBIA, BF0331 of *Bacteroides fragilis* NCTC 9343 and BVU_0115 of *B. vulgatus* ATCC 8482) (Table S2). RT-PCR was conducted in the TechnoSuruga Laboratory using total DNA extracted from the faeces of the two men in their 20’s and 40’s as a template and primers for 16S rRNA or the *Bacteroides* UGL gene with Illumina HiSeq T. The primers (341f and 534r) for the 16S rRNA gene are shown in Table S2. In the same way, RT-PCR was performed using genomic DNA from *B*. *vulgatus* as a positive control. The gene fragments amplified by RT-PCR were subcloned to a pGEM-T Easy Vector System (Promega) and each of clones from the two men was subjected to a determination of nucleotide sequence by the ABI PRISM 3130xl Genetic Analyzer System (Applied Biosystems), as previously described^33^.

### Accession codes

The draft genome sequence (108 contigs) of *E. faecium* strain H57 was deposited in the GenBank/EMBL/DDBJ databases under accession numbers BEHC01000001-BEHC01000108.

## Electronic supplementary material

Supplementary Information
