# Supplementary material for: Probiotics in human gut microbiota can degrade host glycosaminoglycans
Source: Sci Rep. 2018 Jul 13;8:10674. doi: 10.1038/s41598-018-28886-w (PMC6045597; doi:10.1038/s41598-018-28886-w)
Supplement: Supplementary file 1 — Supplementary Information [file 41598_2018_28886_MOESM1_ESM.docx]

**Supplementary Information**

**Probiotics in human gut microbiota can degrade host glycosaminoglycans**

Keigo Kawai^1^, Reiko Kamochi^2^, Sayoko Oiki^1^, Kousaku Murata^3^ & Wataru Hashimoto^1,2^

^1^Laboratory of Basic and Applied Molecular Biotechnology, Division of Food Science and Biotechnology, Graduate School of Agriculture, Kyoto University, Uji, Kyoto 611-0011, Japan. ^2^Laboratory of Basic and Applied Molecular Biotechnology, Department of Food Science and Biotechnology, Faculty of Agriculture, Kyoto University, Uji, Kyoto 611-0011, Japan. ^3^Laboratory of Food Microbiology, Department of Life Science, Faculty of Science and Engineering, Setsunan University, Neyagawa, Osaka 572-8508, Japan. Correspondence and requests for materials should be addressed to W. H. (email: whasimot@kais.kyoto-u.ac.jp)

**Methods**

**GAG assay.** A metachromatic thiazine agent 1,9-dimethylmethylene blue (DMMB) forms a complex with sulfated GAGs like heparin and chondroitin sulfate^1–3^. This complex shows a maximum absorbance at around 525 nm. DMMB reagent was prepared as follows: glycine (0.304 g) and NaCl (0.16 g) were dissolved in 80 ml of pure water. After the addition of 0.1 M acetic acid (9.5 ml) and DMMB (0.016 mg), the solution was adjusted to 100 ml with pure water. The culture supernatant (80 µl) was mixed with the DMMB reagent (800 µl) and the absorbance of the mixture was measured at 525 nm. GAG concentration in the culture supernatant was determined based on the calibration curve prepared by standard heparin or chondroitin sulfate C (0, 10, 20, 30, 40, and 50 µg/ml). The experiments were performed in triplicate. Degradation of GAGs was also analysed by TLC. The culture supernatant (1 µl) was spotted on the TLC plate (silica gel 60 F_254_, Merck) and developed with a solvent system (butanol:acetic acid:pure water = 3:2:2). Sugars were detected by heating the TLC plate at 110°C after spraying it with 10% (v/v) sulfuric acid in ethanol. As polysaccharides are distinct from mono- or oligosaccharides, GAG (heparin or chondroitin sulfate C) was observed at the original spot point without a migration on the TLC plate.

**Draft genome sequence analysis.** Genomic DNA was extracted from strain H57 cells with a NucleoSpin Tissue kit (MACHEREY-NAGEL). The draft genome sequence of strain H57 cells was determined by Hokkaido System Science by a next-generation sequencing system (Illumina HiSeq). A homology search was conducted on the draft sequence by using the Local BLAST programme in BioEdit (Ibis Therapeutics) to search homologous genes involved in the degradation and metabolism of GAGs.

**Expression and purification of KduI and KduD.** Streptococcal DhuI and DhuD involved in the metabolism of DHU from GAGs were purified from recombinant *E*. *coli* BL21(DE3)/pET21b-DhuI and *E*. *coli* BL21(DE3)/pET21b-DhuD, as described previously^4^. The overexpression system of KduI and KduD derived from *L*. *rhamnosus* NBRC 3425 was constructed as follows. DNA manipulations, such as subcloning, transformation, and gel electrophoresis were performed as previously described^5^. The KduI and KduD genes were amplified by PCR using *L*. *rhamnosus* NBRC 3425 cells as a template. The reaction mixture (10 µl) consisted of 0.2 units of DNA polymerase (KOD FX Neo, Toyobo), 0.3 pmol of forward and reverse primers, 4 µmol dNTPs, and a reaction buffer for KOD FX Neo polymerase. Restriction sites of *Nde*I and *Xho*I were added to the 5’ terminus of the forward primer and the 3’ terminus of the reverse primer, respectively (Table S2). The PCR condition was composed of 1 cycle at 94°C for 2 min and the following 30 cycles at 98°C for 10 s, 50°C for 30 s, and 68°C for 1 min. The resultant PCR products were ligated with *Hin*cII-digested pUC119 (Takara Bio). These plasmids (pUC119-KduI and pUC119-KduD) were digested with *Nde*I and *Xho*I to obtain KduI and KduD genes. Each gene was ligated with *Nde*I and *Xho*I-digested pET21b (Novagen). The resultant plasmids (pET21b-KduI and pET21b-KduD) were used to transform *E*. *coli* BL21(DE3) host cells.

Each of the transformant strains, *E*. *coli* BL21(DE3)/pET21b-KduI and BL21(DE3)/pET21b-KduD, was precultured at 37°C and 100 strokes per min (spm) in 10 ml of LB medium containing 0.1 mg/ml sodium ampicillin for 1 d. *E*. *coli* cells (10 ml) were transferred into fresh LB medium (1.5 l/each strain) and cultured at 37°C and 100 spm. When the culture broth showed an OD_600_ of around 0.4, isopropyl-β-D-1-thiogalactopyranoside was added to the culture broth at a final concentration of 0.1 mM and further cultivation was carried out at 16°C and 100 spm for 48 h. *E*. *coli* cells were harvested by centrifugation at 11,200 × g and 4°C for 10 min, resuspended in 25 ml of 20 mM Tris-HCl (pH 7.5), and ultrasonically disrupted at 0°C and 9 kHz for 20 min (Insonator Model 201M, Kubota). After centrifugation at 17,418 × g and 4°C for 20 min, the supernatant was used as the cell extract.

Each cell extract was mixed with 10 ml of TALON Metal Affinity Resin (Clontech) and the mixture was rotated at 6°C overnight to combine the target protein with the resin. After rotation, the resin was recovered as a precipitant by centrifugation of the mixture at 178 × g and 4°C for 5 min and washed with an equilibrium buffer [20 mM Tris-HCl (pH 7.5) containing 0.15 M NaCl and 10 mM imidazole] twice. After washing, the resin was packed into the column. Absorbed proteins were eluted as 10 fractions (3 ml/each) from the resin with 30 ml of an elution buffer [20 mM Tris-HCl (pH 7.5) containing 0.15 M NaCl and 1 M imidazole]. Each fraction was subjected to sodium dodecyl sulfate (SDS)-polyacrylamide gel electrophoresis (PAGE), followed by staining with coomassie brilliant blue^6^. The fractions containing the purified KduI or KduD were dialysed at 4°C for 3 h against 1 l of 20 mM Tris-HCl (pH 7.5) and the dialysate was subjected to the enzyme assay.

**Enzyme assay.** Unsaturated uronic acids from GAGs have been found to be metabolised through successive reactions of isomerase DhuI and NADH-dependent reductase DhuD^4^. Accompanying the enzyme reaction, NADH is oxidised by DhuD and absorbance at 340 nm derived from NADH decreases. The purified KduI and KduD were subjected to this enzyme assay. Briefly, the reaction solution was prepared by mixing 0.5 mM unsaturated chondroitin disaccharide with a sulfate group at the C6 position of *N*-acetyl-D-galactosamine (SEIKAGAKU Biobusiness), the purified UGL of *S*. *agalactiae*^7^, isomerase (DhuI^4^ or KduI), and reductase (DhuD^4^ or KduD) in 20 mM Tris-HCl (pH 7.5). After the addition of NADH at a final concentration of 0.2 mM, the absorbance at 340 nm of the reaction mixture (500 µl) in the cuvette was immediately monitored at 30°C.

**References**

1. Farndale, R. W., Sayers, C. A. & Barrett, A. J. A direct spectrophotometric microassay for sulfated glycosaminoglycans in cartilage cultures. *Connect. Tissue Res.* **9,** 247-248 (1982).

2. Chandrasekhar, S., Esterman, M. A. & Hoffman, H. A. Microdetermination of proteoglycans and glycosaminoglycans in the presence of guanidine hydrochloride. *Anal. Biochem.* **161,** 103-108 (1987).

3. Whitley, C. B., Ridnour, M. D., Draper, K. A., Dutton, C. M. & Neglia, J. P. Diagnostic-test for mucopolysaccharidosis. 1. Direct method for quantifying excessive urinary glycosaminoglycan excretion. *Clin. Chem.* **35,** 374-379 (1989).

4. Maruyama, Y., Oiki, S., Takase, R., Mikami, B., Murata, K. & Hashimoto, W. Metabolic fate of unsaturated glucuronic/iduronic acids from glycosaminoglycans molecular identification and structure determination of streptococcal isomerase and dehydrogenase. *J. Biol. Chem.* **290**, 6281-6292 (2015).

5. Sambrook, J., Fritsch, E. F. & Maniatis, T. Molecular Cloning. A Laboratory Manual, 2nd ed. Cold Spring Harbor Laboratory Press, Cold Spring Harbor, N.Y. (1989).

6. Laemmli, U. K. Cleavage of structural proteins during the assembly of the head of bacteriophage T4. *Nature* **227,** 680-685 (1970).

7. Maruyama, Y., Nakamichi, Y., Itoh, T., Mikami, B., Hashimoto, W. & Murata, K. Substrate specificity of streptococcal unsaturated glucuronyl hydrolases for sulfated glycosaminoglycan. *J. Biol. Chem.* **284**, 18059-18069 (2009).

**Table S1.** Draft genome sequence of *E. faecium* strain H57.

| Total Contig length (bp) | | 2,583,114 | |
| --- | --- | --- | --- |
| # contigs | | 108 | |
| N50 (bp) | | 73,437 | |
| N90 (bp) | | 18,565 | |
| Max Contig length (bp) | | 175,910 | |
| **Accesion No.** | **Contig_Name** | **Length** | **GC (％)** |
| BEHC01000001 | NODE_1_length_64762_cov_24.514475 | 64,762 | 38.19 |
| BEHC01000002 | NODE_2_length_95370_cov_25.907164 | 95,370 | 38.72 |
| BEHC01000003 | NODE_3_length_1098_cov_306.762939 | 1,098 | 36.52 |
| BEHC01000004 | NODE_4_length_39377_cov_20.425171 | 39,377 | 34.62 |
| BEHC01000005 | NODE_5_length_112570_cov_22.748320 | 112,570 | 38.13 |
| BEHC01000006 | NODE_6_length_64537_cov_30.205748 | 64,537 | 38.53 |
| BEHC01000007 | NODE_7_length_115499_cov_24.215683 | 115,499 | 37.53 |
| BEHC01000008 | NODE_8_length_16840_cov_20.229069 | 16,840 | 36.10 |
| BEHC01000009 | NODE_9_length_68038_cov_27.398167 | 68,038 | 37.69 |
| BEHC01000010 | NODE_10_length_157552_cov_26.719423 | 157,552 | 38.03 |
| BEHC01000011 | NODE_11_length_53837_cov_26.114155 | 53,837 | 37.65 |
| BEHC01000012 | NODE_12_length_7689_cov_70.587883 | 7,689 | 38.50 |
| BEHC01000013 | NODE_13_length_1546_cov_134.756195 | 1,546 | 52.26 |
| BEHC01000014 | NODE_14_length_101810_cov_26.783348 | 101,810 | 38.36 |
| BEHC01000015 | NODE_15_length_93554_cov_22.308817 | 93,554 | 38.47 |
| BEHC01000016 | NODE_16_length_22818_cov_30.331017 | 22,818 | 39.20 |
| BEHC01000017 | NODE_17_length_38725_cov_30.189951 | 38,725 | 38.41 |
| BEHC01000018 | NODE_18_length_47983_cov_25.182234 | 47,983 | 38.42 |
| BEHC01000019 | NODE_19_length_12280_cov_28.124815 | 12,280 | 34.81 |
| BEHC01000020 | NODE_20_length_5303_cov_28.023613 | 5,303 | 32.08 |
| BEHC01000021 | NODE_21_length_32521_cov_24.598667 | 32,521 | 37.92 |
| BEHC01000022 | NODE_22_length_18078_cov_26.518906 | 18,078 | 36.05 |
| BEHC01000023 | NODE_23_length_208_cov_242.280701 | 208 | 49.52 |
| BEHC01000024 | NODE_24_length_63050_cov_28.862015 | 63,050 | 39.17 |
| BEHC01000025 | NODE_25_length_56948_cov_21.985189 | 56,948 | 38.07 |
| BEHC01000026 | NODE_26_length_5204_cov_30.187475 | 5,204 | 32.21 |
| BEHC01000027 | NODE_27_length_569_cov_71.048424 | 569 | 41.48 |
| BEHC01000028 | NODE_28_length_15540_cov_26.149424 | 15,540 | 39.00 |
| BEHC01000029 | NODE_29_length_34794_cov_28.503977 | 34,794 | 39.23 |
| BEHC01000030 | NODE_30_length_1858_cov_23.147959 | 1,858 | 32.45 |
| BEHC01000031 | NODE_31_length_71898_cov_27.307949 | 71,898 | 38.08 |
| BEHC01000032 | NODE_32_length_60961_cov_20.763008 | 60,961 | 38.29 |
| BEHC01000033 | NODE_33_length_704_cov_45.303280 | 704 | 52.56 |
| BEHC01000034 | NODE_34_length_23649_cov_26.262661 | 23,649 | 38.32 |
| BEHC01000035 | NODE_35_length_127769_cov_28.448553 | 127,769 | 39.47 |
| BEHC01000036 | NODE_36_length_15521_cov_25.690737 | 15,521 | 39.73 |
| BEHC01000037 | NODE_37_length_35477_cov_22.759348 | 35,477 | 39.11 |
| BEHC01000038 | NODE_39_length_221_cov_103.181099 | 221 | 37.10 |
| BEHC01000039 | NODE_40_length_33281_cov_22.931299 | 33,281 | 37.03 |
| BEHC01000040 | NODE_41_length_4131_cov_21.901411 | 4,131 | 36.46 |
| BEHC01000041 | NODE_42_length_24592_cov_23.610376 | 24,592 | 34.76 |
| BEHC01000042 | NODE_44_length_32688_cov_25.038473 | 32,688 | 38.09 |
| BEHC01000043 | NODE_45_length_26062_cov_21.028997 | 26,062 | 36.54 |
| BEHC01000044 | NODE_46_length_73437_cov_21.160915 | 73,437 | 38.34 |
| BEHC01000045 | NODE_47_length_15722_cov_20.885462 | 15,722 | 37.95 |
| BEHC01000046 | NODE_49_length_8844_cov_25.626858 | 8,844 | 37.16 |
| BEHC01000047 | NODE_50_length_18565_cov_24.015593 | 18,565 | 37.54 |
| BEHC01000048 | NODE_51_length_204_cov_43.945454 | 204 | 38.24 |
| BEHC01000049 | NODE_52_length_8750_cov_29.271141 | 8,750 | 38.69 |
| BEHC01000050 | NODE_53_length_11938_cov_21.505825 | 11,938 | 34.91 |
| BEHC01000051 | NODE_54_length_7474_cov_22.186857 | 7,474 | 37.08 |
| BEHC01000052 | NODE_55_length_31241_cov_20.631649 | 31,241 | 37.80 |
| BEHC01000053 | NODE_56_length_690_cov_207.211411 | 690 | 34.49 |
| BEHC01000054 | NODE_57_length_10937_cov_27.693350 | 10,937 | 39.74 |
| BEHC01000055 | NODE_58_length_2838_cov_25.312317 | 2,838 | 30.51 |
| BEHC01000056 | NODE_59_length_175910_cov_24.143400 | 175,910 | 38.59 |
| BEHC01000057 | NODE_60_length_8453_cov_28.284962 | 8,453 | 41.46 |
| BEHC01000058 | NODE_61_length_729_cov_236.017319 | 729 | 34.84 |
| BEHC01000059 | NODE_62_length_542_cov_130.602676 | 542 | 35.98 |
| BEHC01000060 | NODE_64_length_1008_cov_128.472641 | 1,008 | 34.82 |
| BEHC01000061 | NODE_65_length_2120_cov_20.015795 | 2,120 | 44.29 |
| BEHC01000062 | NODE_66_length_1679_cov_51.255520 | 1,679 | 38.06 |
| BEHC01000063 | NODE_67_length_462_cov_25.932066 | 462 | 39.39 |
| BEHC01000064 | NODE_68_length_7648_cov_26.260656 | 7,648 | 34.68 |
| BEHC01000065 | NODE_69_length_5928_cov_21.253342 | 5,928 | 39.66 |
| BEHC01000066 | NODE_70_length_3174_cov_32.735714 | 3,174 | 30.28 |
| BEHC01000067 | NODE_71_length_20734_cov_22.429214 | 20,734 | 37.51 |
| BEHC01000068 | NODE_72_length_92090_cov_20.115667 | 92,090 | 39.07 |
| BEHC01000069 | NODE_73_length_37927_cov_24.160442 | 37,927 | 38.28 |
| BEHC01000070 | NODE_74_length_664_cov_141.357895 | 664 | 38.70 |
| BEHC01000071 | NODE_78_length_1062_cov_265.771698 | 1,062 | 35.40 |
| BEHC01000072 | NODE_79_length_74585_cov_20.605120 | 74,585 | 37.67 |
| BEHC01000073 | NODE_80_length_1337_cov_53.438454 | 1,337 | 42.41 |
| BEHC01000074 | NODE_81_length_278_cov_65.000000 | 278 | 39.21 |
| BEHC01000075 | NODE_82_length_810_cov_79.513969 | 810 | 36.54 |
| BEHC01000076 | NODE_83_length_2561_cov_20.987839 | 2,561 | 30.73 |
| BEHC01000077 | NODE_84_length_258_cov_66.932930 | 258 | 35.27 |
| BEHC01000078 | NODE_85_length_27403_cov_22.908272 | 27,403 | 36.47 |
| BEHC01000079 | NODE_86_length_252_cov_98.651901 | 252 | 34.13 |
| BEHC01000080 | NODE_88_length_290_cov_46.811226 | 290 | 41.72 |
| BEHC01000081 | NODE_89_length_342_cov_27.604839 | 342 | 32.75 |
| BEHC01000082 | NODE_90_length_399_cov_21.308197 | 399 | 31.58 |
| BEHC01000083 | NODE_92_length_75561_cov_27.496746 | 75,561 | 39.55 |
| BEHC01000084 | NODE_93_length_1848_cov_30.081528 | 1,848 | 33.50 |
| BEHC01000085 | NODE_94_length_220_cov_25.182539 | 220 | 30.00 |
| BEHC01000086 | NODE_95_length_240_cov_46.821918 | 240 | 28.75 |
| BEHC01000087 | NODE_96_length_1012_cov_69.521790 | 1,012 | 40.02 |
| BEHC01000088 | NODE_97_length_685_cov_25.340101 | 685 | 33.72 |
| BEHC01000089 | NODE_98_length_14448_cov_28.496586 | 14,448 | 35.67 |
| BEHC01000090 | NODE_100_length_203_cov_23.504587 | 203 | 36.95 |
| BEHC01000091 | NODE_102_length_534_cov_50.770454 | 534 | 43.07 |
| BEHC01000092 | NODE_105_length_194_cov_52.770000 | 194 | 49.48 |
| BEHC01000093 | NODE_106_length_257_cov_74.907974 | 257 | 35.80 |
| BEHC01000094 | NODE_107_length_203_cov_122.376144 | 203 | 35.96 |
| BEHC01000095 | NODE_108_length_316_cov_25.256756 | 316 | 25.95 |
| BEHC01000096 | NODE_109_length_201_cov_26.542055 | 201 | 34.83 |
| BEHC01000097 | NODE_111_length_215_cov_68.082642 | 215 | 44.65 |
| BEHC01000098 | NODE_112_length_758_cov_43.859940 | 758 | 40.77 |
| BEHC01000099 | NODE_113_length_529_cov_98.641380 | 529 | 37.05 |
| BEHC01000100 | NODE_115_length_230_cov_24.529411 | 230 | 29.13 |
| BEHC01000101 | NODE_116_length_218_cov_43.637096 | 218 | 38.07 |
| BEHC01000102 | NODE_118_length_14365_cov_26.813328 | 14,365 | 38.36 |
| BEHC01000103 | NODE_121_length_208_cov_41.035088 | 208 | 49.04 |
| BEHC01000104 | NODE_122_length_573_cov_147.106476 | 573 | 36.65 |
| BEHC01000105 | NODE_124_length_3237_cov_136.317856 | 3,237 | 49.77 |
| BEHC01000106 | NODE_154_length_217_cov_37.357723 | 217 | 45.16 |
| BEHC01000107 | NODE_160_length_252_cov_50.088608 | 252 | 35.32 |
| BEHC01000108 | NODE_173_length_193_cov_101.858589 | 193 | 36.27 |

**Table S2.** List of primers used in this study.

| Primer | Sequence |
| --- | --- |
| 27f for 16S rRNA | 5’- AGAGTTTGATCMTGGCTCAG-3’ |
| 1492r for 16S rRNA | 5’- TACGGYTACCTTGTTACGACTT-3’ |
| Forward for *kduI* | 5’-CATATGAGTTTTAGCATGGTCACACGTT-3’ |
| Reverse for *kduI* | 5’-CTCGAGGCGTAATTCATTCATTGCTACTTGA-3’ |
| Forward for *kduD* | 5’-CATATGAGTGAAATTCACCAAACTGAAG-3’ |
| Reverse for *kduD* | 5’-CTCGAGGCTAACAAGGAAACCACCGTCAACA-3’ |
| Forward for *Bacteroides ugl* | 5’-ATBGAYAAYATGATSAAYCTSGA-3’ |
| Reverse for *Bacteroides ugl* | 5’-GCYTGHCCRCGWGMCCA-3’ |
| 341f for 16S rRNA | 5’-CCTACGGGAGGCAGCAG-3’ |
| 534r for 16S rRNA | 5’-ATTACCGCGGCTGCTGG-3’ |


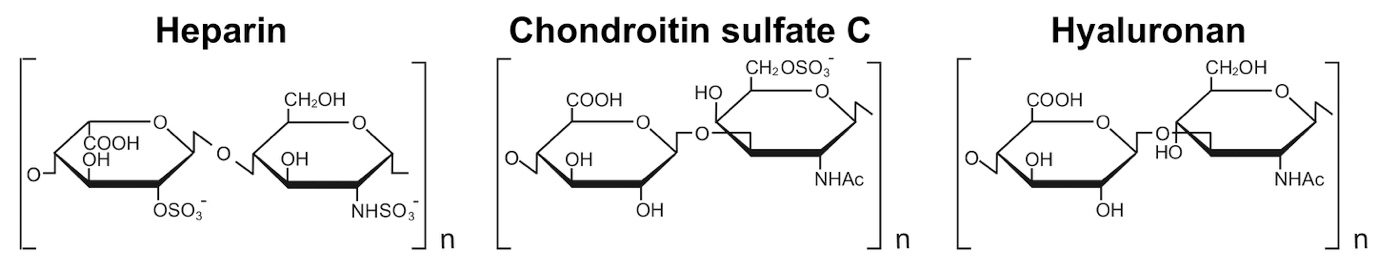


**Figure S1.** Structure of GAGs (typical heparin, chondroitin sulfate C and hyaluronan).


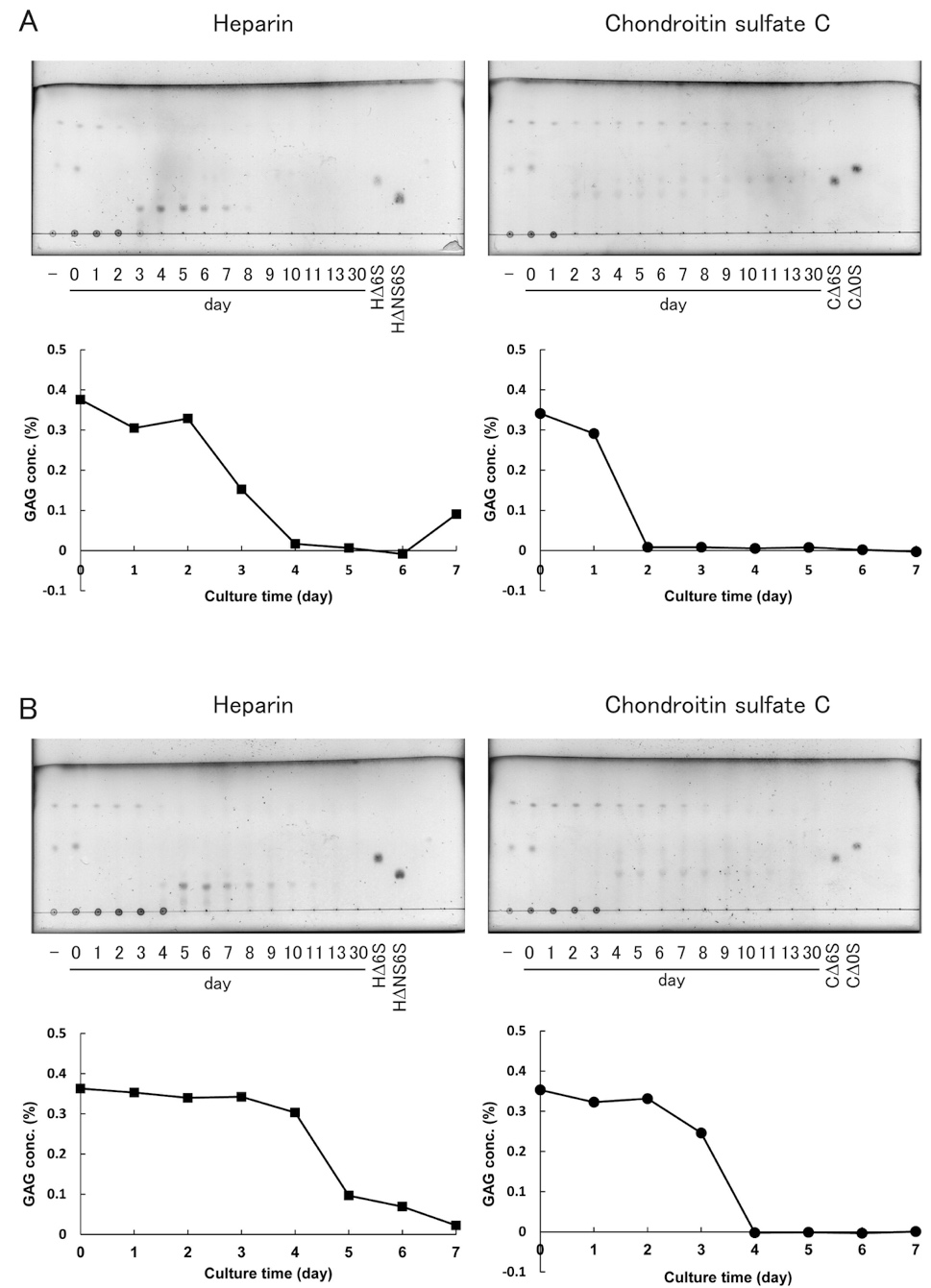


**Figure S2.** Degradation profiles of GAGs by human gut microbiota. Gut microbiota from two human faeces were co-cultured for 30d in the nutrition medium containing heparin (left) and chondroitin sulfate C (right). The supernatants derived from periodically sampled culture broth were subjected to TLC (upper) and GAG assays (lower). (**A**) Japanese man in his 50’s. (**B**) Japanese woman in her 20’s. GAGs were depolymerised through generation of probable disaccharides as an intermediate and finally completely degraded as seen in sample at 30d. These profiles are not an image cropped from different parts of the same TLC plate or from different TLC plates.


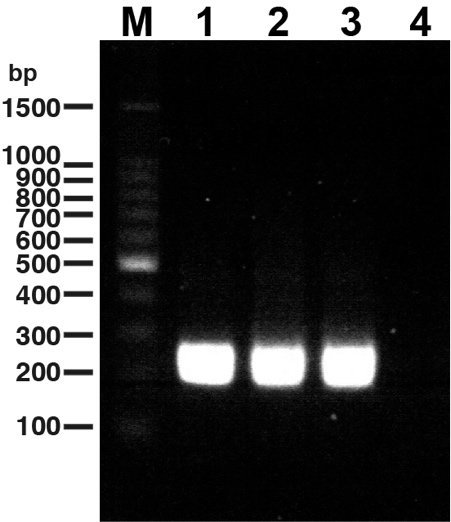


**Figure S3.** RT-PCR for detection of the *Bacteroides* UGL gene in human gut microbiota. The enzyme gene was readily found to be included in the gut microbiota of Japanese men in their 20’s and 40’s. Lanes are explained by the template used in RT-PCR. Lane M, markers of the 100 bp ladder; lane 1, DNA from *B*. *vulgatus*; lane 2, faecal DNA from the man in his 40’s; lane 3, faecal DNA from the man in his 20’s; and lane 4, without a template. This electrophoretic profile is not an image cropped from different parts of the same gel or from different gels.
